# Vitamins A and D and Zinc Affect the Leshmanicidal Activity of Canine Spleen Leukocytes

**DOI:** 10.3390/ani11092556

**Published:** 2021-08-31

**Authors:** Fabiana M. de O. Hernandez, Marilene O. Santos, Gabriela L. Venturin, Jaqueline P. Bragato, Gabriela T. Rebech, Larissa M. Melo, Sidnei F. Costa, Jéssica H. de Freitas, Carlos Eduardo Siqueira, Déborah A. Morais, Wellington T. de S. Júnior, Fernando B. Júnior, Flávia L. Lopes, Valéria M. F. de Lima

**Affiliations:** 1 Department of Clinical Medicine, Surgery and Animal Reproduction, School of Veterinary Medicine, São Paulo State University (UNESP), Araçatuba 16050-680, SP, Brazil; fabi-nut@hotmail.com (F.M.d.O.H.); marilene.bio.ata@gmail.com (M.O.S.); gabriela_venturin@hotmail.com (G.L.V.); jaquelinepoleto@hotmail.com (J.P.B.); gabriela.t.rebech@unesp.br (G.T.R.); lala_mmelo@yahoo.com.br (L.M.M.); sidnei.f.costa@unesp.br (S.F.C.); jessica_hdf@yahoo.com.br (J.H.d.F.); carlos.siqueira@unesp.br (C.E.S.); 2 Toxicology and Metals Essentiality Department, School of Pharmaceutical Scienses, Sao Paulo University (USP), Ribeirão Preto 14040-903, SP, Brazil; debymorais11@gmail.com (D.A.M.); wellingtonjunior123@hotmail.com (W.T.d.S.J.); fbarbosa@fcfrp.usp.br (F.B.J.); 3 Clinics Department Production and Animal Health, School of Veterinary Medicine, São Paulo State University (UNESP), Araçatuba 16050-680, SP, Brazil; flavia.lopes@unesp.br

**Keywords:** retinol, all-trans retinoic acid, vitamin D3, zinc, *Leishmania* spp.

## Abstract

**Simple Summary:**

Canine leishmaniasis (CanL) is a zoonosis caused by the protozoan *Leishmania infantum*, with dogs being considered the main domestic reservoirs of the parasite and potential transmitters to humans through the phlebotomine vector. CanL is a chronic infectious disease that characteristically occurs in underdeveloped and developing countries, with a broad spectrum of clinical and immunopathological manifestations. Despite the efforts in the fight against the disease, many endemic sites of CanL persist, preceding human infection and representing a serious public health problem. The therapeutic arsenal for the treatment of leishmaniasis is limited by toxicity, high costs, and inefficacy, in some cases. Treatment failure implies the permanence of dogs as reservoirs of the parasite, with further aggravation of the public health problem, indicating that new therapies should be evaluated in order to increase the treatment efficacy. Recent studies have shown that nutrients can regulate the immune response in many clinical conditions, but no study has been conducted using spleen leukocytes in CanL. In this paper, we demonstrate for the first time that nutrients added to cultures of spleen leukocytes from dogs infected with CanL can modulate the immune response and parasite load compared to healthy dogs.

**Abstract:**

Canine leishmaniasis (CanL) is a chronic disease caused by *Leishmania infantum*, and the limitations of the current treatments have encouraged new alternatives, such as the use of immunomodulatory nutrients. The objective of this study was to determine the serum levels of vitamin A (retinol), vitamin D (25(OH)VD_3_), and zinc (Zn) in dogs with CanL and the effect of in vitro supplementation with the respective active forms ATRA, 1,25(OH)_2_VD_3_, and SZn on spleen leukocyte cultures. Serum retinol, 25(OH)VD_3_, and Zn were determined by HPLC, ELISA, and ICP-MS, respectively. Spleen leukocyte cultures were used for the detection of NO and ROS by flow cytometry; the IFN-γ, TNF-α, and IL-10 levels were determined by ELISA; and the parasite load was determined by microscopy. We detected low serum levels of retinol and Zn and high levels of 25(OH)VD_3_ in the CanL group. The in vitro supplementation of CanL spleen leukocytes with ATRA, 1,25(OH)_2_VD_3_, and SZn, in addition to a soluble leishmania antigen (SLA) treatment, increased the NO and ROS levels, while the treatments with only ATRA and SZn increased the TNF-a levels. Increased IL-10 and IFN-g levels were observed with the addition of SLA to the medium, although the addition of the three nutrients led to a reduction of the IL-10 levels, and the addition of 1,25(OH)_2_VD_3_ and SZn led to a reduction of IFN-g. A supplementation with 1,25(OH)_2_VD_3_ and SZn reduced the parasite load but only in the absence of SLA. We suggest that the nutrients we tested are involved in the leishmanicidal mechanism, showing a potential for investigation in future studies.

## 1. Introduction

Canine leishmaniasis (CanL) is a chronic infectious disease that characteristically occurs in underdeveloped and developing countries. It is caused by *Leishmania* spp., such as *L. infantum*, with a broad spectrum of clinical and immunopathological manifestations. Dogs are considered the main reservoirs of the parasite in urban centers, and, despite efforts made in the fight against the disease, many endemic sites of canine leishmaniasis (CanL) persist, preceding human infection and representing a serious public health problem [1].

It has been well-established that one of the most important aspects of the pathogenesis and progression of CanL is the suppression of Th1 cell immunity, with a predominance of Th2 cell immunity, which is ineffective in the elimination of the parasite [2]. The involvement of regulatory T cells in suppressing the immune response is also clear, as demonstrated by the production of interleukin 10 (IL-10) by CD4+FOXP3+T cells in CanL spleens [3]. The absence of a T-cell response to *Leishmania* spp. antigens has been observed in infected dogs based on negative skin tests. However, a Th1 cell effector response in the fight against the parasite is detected in resistant animals [2,4]. Previous studies have suggested the addition of soluble leishmania antigen (SLA) stimulation as an immunological marker for the induction of Th1 in vitro studies [5,6]. The Th1 cell effector response is characterized by the action of TCD4+ lymphocytes, which secrete Th1 type cytokines such as interferon gamma (IFN-γ) and tumor necrosis factor (TNF-α). By contrast, humoral immunity is characterized by the secretion of type Th2 cytokines, such as IL-10 (also from Treg cells), and is associated with parasite survival and disease persistence [7,8].

The role of reactive species, such as nitric oxide (NO) and reactive oxygen species (ROS), is also important for leishmanicidal activity, especially by macrophages, which are considered the main cell type infected by *Leishmania* spp. parasites [9]. ROS are generated during the initial infection as part of the oxidative explosion [10], while NO is generated after the macrophage activation by IFN-γ and TNF-α [11,12].

The therapeutic arsenal for the treatment of leishmaniasis is limited by toxicity, high costs, and, in some cases, inefficacy [13,14]. Treatment failure implies the persistence of dogs as a reservoir of the parasite, with further aggravation of the public health problem, indicating that new therapies should be evaluated in order to increase the treatment efficacy. The relationship between the disease, nutrition, and immunity, although not yet fully elucidated, has been recognized for many years and is currently being investigated under different clinical condition experimental models. Nutrients are necessary for immune system functions [15]. They may act synergistically with drug treatments [16] and can help prevent a series of infectious contagious diseases [17].

The importance of nutrients for the regulation of immunity has seldom been studied in visceral leishmaniasis. Zinc (Zn) is an essential mineral for innate and adaptive immune responses [18]. In dogs, Zn supplementation in combination with standard therapy has elicited a more rapid therapeutic response and a delay in relapse [16], while a vitamin D deficiency is associated with disease progression [19]. In patients with visceral leishmaniasis, serum vitamin A has been found to be reduced, and an in vitro supplementation with vitamin A in combination with SLA stimulus promoted a reduction of IL-10 in Treg cells and monocytes [20]. In mice infected with *L. donovani*, a vitamin A and vitamin D supplementation improved the immunity and reduced the parasite load in the spleen [21]. 

Despite the evidence of the role of nutrients in the immune system and in leishmaniasis, few studies have been conducted on dogs. Thus, in this study, our objective was to determine the serum levels of vitamin A (retinol), vitamin D (25-hydroxy vitamin D3 or 25OHVD_3_), and Zn in dogs with visceral leishmaniasis and to assess in vitro the ability of these nutrients to regulate the production of nitric oxide (NO), reactive oxygen species (ROS), and cytokines; regulate the adaptive responses of IFN-γ, TNF-α, and IL-10; and monitor the parasite load in CanL. This study provides critical data on the complex role of vitamins A and D and Zn in the immune system, specifically in relation to CanL, and the innate response of splenic leukocytes, which will help inform future in vivo studies.

## 2. Materials and Methods

### 2.1. Dogs and Sample Collection

This study was approved by the Ethics Committee for the Use of Animals of Sao Paulo State University (Unesp), School of Veterinary Medicine Araçatuba (Protocol N° 00165-2017). The group of dogs with leishmaniasis (CanL) consisted of 15 adult animals (8 male and 7 female) who were seropositive for the *L. infantum* antigen, as determined by an indirect ELISA [22], with the diagnosis confirmed by the detection of *Leishmania* sp. DNA by real-time PCR (qPCR) [23]. The dogs were symptomatic and exhibited at least three of the following clinical signs: onychogryphosis, cachexia, alopecia, skin and periocular lesions, and lympho-hepatosplenomegaly. Animals were obtained from the Center of Zoonosis Control, aged 2–5 years old and varied in breed and weight, with the blood count and biochemical parameters expected as moderate disease stages, according to the parameters proposed by Solano and Galego [14]. The control group consisted of 5 healthy dogs (2 male and 3 female) that resided in an endemic area but tested negative for *Leishmania* spp., as determined by indirect ELISA and qPCR, with the blood count and biochemical parameters within the normal range for the species.

The blood samples were collected by puncture of the jugular vein and placed in dry tubes, with the serum obtained immediately for the later determination of the vitamins A and D and Zn levels. Spleen tissue samples were obtained from the CanL group after euthanasia was performed according to the state legislation with an intravenous injection of a barbituric anesthetic (thiopental, Cristália Itapira, SP), followed by a 19.1% solution of potassium chloride. A spleen fragment was obtained from healthy dogs by surgical excision, according to the protocol described by Lima et al. [2].

### 2.2. Real-Time PCR

*Leishmania* sp. DNA was detected in the peripheral blood samples by qPCR on a Mastercycler-Ep Realplex 4-S (Eppendorf North America, Westbury, NY, USA) using the ITS1 primer (5′AGCTGGATCATTTTCCGATG3′ and 5′TATGTGAGCCGTTATCCACGC3′) according to the protocol described by Perosso et al. [23].

### 2.3. Serum Micronutrient Assays

The vitamin A serum was determined based on its circulating form (retinol) by HPLC using a 4.6 cm × 25 cm C-18 column (Shimpack CLC-ODS), a 4 mm × 1 cm precolumn, and a flow of 2.0 mL/min [24]. 

The vitamin D serum was determined based on its circulation form (25(OH)VD_3_) by competitive capture ELISA using the commercial Dog 25-hydroxy vitamin D3 kit (BiorByt, Cambridge, UK) according to the manufacturer’s instructions. 

The Zn levels were determined in the serum samples with an inductively coupled plasma mass spectrometer (PerkinElmer, NexION 2000 B, Waltham, MA, USA, EUA). The analysis was performed as described by Batista et al. [25]. Briefly, the samples (0.1 mL) were directly diluted in a metal-free polypropylene Falcon tube (Becton Dickinson, Franklin Lakes, NJ, USA) containing 4.9 mL of the diluent solution composed of 0.005% *v*/*v* Triton^®^ X-100 and 0.5% *v*/*v* distilled HNO_3_. The dilutions were directly injected for ICP-MS measurements. The analytical calibration standards were prepared daily over the range of 10–100 μg L^–1^.

### 2.4. Cell Culture

Whole spleen cells were obtained from a 3 cm^3^ macerated fragment and added to 10-mL RPMI-1640 medium supplemented with fetal bovine serum inactivated to 10% (Gibco, Waltham, MA, USA), 0.03% L-glutamine (Sigma, St. Louis, MO, USA), 100-IU/mL penicillin (Sigma), and 100-μg/mL streptomycin (Sigma). After the removal of cell debris through a BD Falcon Cell filter (San Diego, CA, USA), the cell suspension was processed with 5-mL erythrocyte lysis buffer containing 7.46-g/L ammonium chloride (NH_4_ClO_3_) at 4 °C for 10 min, centrifuged at 2000 rpm for 5 min, and washed three times with phosphate-buffered saline (pH 7.2). 

Spleen leukocyte cultures composed mainly of lymphocytes, macrophages, dendritic cells, and plasma cells, for a total of 2.5 × 10^6^ cells, were counted in a Neubauer chamber with 400 μL per well and incubated in RPMI-1640 medium in an incubator at 37 °C with 5% CO_2_ for 20 h to detect the reactive species and for 72 h to detect the cytokines and measure the parasite load. 

A total of 9 treatment conditions were used in this study: (1) Basal medium or control (RPMI-1640 medium plus cells), (2) basal medium plus the addition of the active form of vitamin A (all-trans retinoic acid or ATRA) at 0.5 nM, (3) basal medium plus the addition of the active form of vitamin D (1,25-dihydroxy vitamin D3 or 1,25(OH)_2_VD_3_) at 4 nM, (4) basal medium plus an addition of zinc sulfate heptahydrate (SZn) at 0.05 nM, (5) basal medium plus an addition of 10-μg/mL soluble leishmania antigen (SLA), (6) basal medium plus an addition of ATRA+SLA, (7) basal medium plus an addition of 1,25(OH)_2_VD_3_+SLA, (8) basal medium plus an addition of SZn+SLA, and (9) basal medium plus an addition of 10-μg/mL phytohemagglutinin (PHA). 

The concentration of SLA and PHA used in the canine spleen leukocyte cultures was based on data from the literature [20]; the SLA was intended to stimulate a specific immune response to the leishmania antigen, and PHA was used as a positive control in the dosage of the cytokines. The nutrients ATRA, 1,25(OH)_2_VD_3_, and SZn were reconstituted in DMSO, ethanol, and RPMI-1640 medium, respectively, according to the manufacturer’s recommendations for use in cell cultures. After reconstitution, the nutrients were successively diluted in the RPMI-1640 medium, from the order of milligrams to nanograms. Three different concentrations for each nutrient were tested in a previous pilot study, based on the literature [20,26,27] and including variations of ±50% of the reference values, and the percentage of cell death by apoptosis was titrated by flow cytometry using the Guava Nexin kit (Millipore) according to the manufacturer’s instructions. In the cytometry analysis, we considered the percentage of cells in late apoptosis that were positive for PE-conjugated annexin and 7AA-D collected from the FL2 and FL3 channels of the flow cytometer, respectively. We chose the concentration that promoted the lowest proportion of cell death by apoptosis. 

### 2.5. Flow Cytometry

The NO and ROS levels in spleen leukocytes were determined after 20 h of cell culture. For ROS determination, the cell suspension was treated and stained with 10-μM H2DCFDA (Invitrogen-Leiden Molecular Probes, Bleiswijk, Lansingerland, The Netherlands) and incubated for 30 min at 37 °C in the presence of 5% CO_2_. For the NO determination, the cell suspension was treated with 2-μM DAF-2DA for 30 min at 37 °C in the presence of 5% CO_2_. Unlabeled samples were used as a negative control to define the negative populations in the samples analyzed. We measured the green fluorescence (excitation = 492–495 nm and emission = 517–527 nm) using an FL1 filter and considered 10,000 closed events. The data were analyzed using BD Accuri C6 software, version 1.0.264 (BD Biosciences, San Jose, CA, USA). 

### 2.6. ELISA

The cytokines IFN-γ, TNF-α, and IL-10 were quantitated in the supernatant of dog leukocytes after 72 h of culture using commercial ELISA capture Duo Set^®^ Canine kits (R&D System, Minneapolis, MN, USA) according to manufacturer’s instructions. Plate readings were obtained with a Spectra Count spectrophotometer (Packard Bio Science Company, Meriden, CT, USA) with a 450-nm filter. 

### 2.7. Parasite Load by Count under a Light Microscope

The parasite load was determined in canine spleen leukocytes after 72 h of incubation by cytocentrifugation at 1000 rpm for 5 min at room temperature. The slides were stained with a commercial hematological dye (Instant-Prov, Newprov, Pinhais, PR, Brazil) for parasite counting inside the macrophages under a light microscope (Eclipse E800, Nikon, Tokyo, Japan) for differentiation of the infected and noninfected cells. We counted 50 infected macrophages (when the infection rate was low, we considered the maximum number of infected macrophages) and the quantity of amastigotes inside the cells. The parasite load was determined by dividing the number of amastigotes by the number of infected macrophages counted. The results are expressed as a percentage (%), considering the medium as 100% in relation to the other treatments.

### 2.8. Reagents and Assays

The vitamins A and D and Zn serums were determined using Retinol (synthetic, ≥95% HPLC, crystalline, Sigma), a commercial Kit Dog 25-hydroxy Vitamin D3 (BiorByt), and Zn (Zinc sulfate concentrate, Sigma), respectively. The DNA was extracted using a commercial DNAeasy (Qiagen) kit. The in vitro cultures were supplemented with vitamin A (all-trans retinoic acid, Biomedicals MP), vitamin D (1α,25-dihydroxyvitamin D3, Sigma), Zn (zinc sulfate heptahydrate, Sigma), and phytohemagglutinin, M (PHA-M, Gibco Thermo Fisher Scientific). NO and ROS were quantitated using a DAF-2DA probe (2 μM) and H2DCFDA probe (10 μM) (Invitrogen), respectively. IFN-γ, TNF-α, and IL-10 were quantitated with commercial Duo Set Canine kits (R&D System).

### 2.9. Statistical Analysis

The data were tested for normality, and the Mann–Whitney test was used to compare the serum retinol, 25(OH)VD_3_, and Zn levels between the CanL group and control group of healthy dogs. A Wilcoxon test was used to compare the production of NO and ROS; cytokines IFN-γ, TNF-α, and IL-10; and the parasite load between treatments. All analyses were carried out and graphs were constructed using GraphPad Prisma 6 software (La Jolla, CA, USA), with the level of statistical significance set at *p* < 0.05.

## 3. Results

### 3.1. Reduction of Retinol and Zn and Increase in 25(OH)VD_3_ Serum

**Figure 1:**
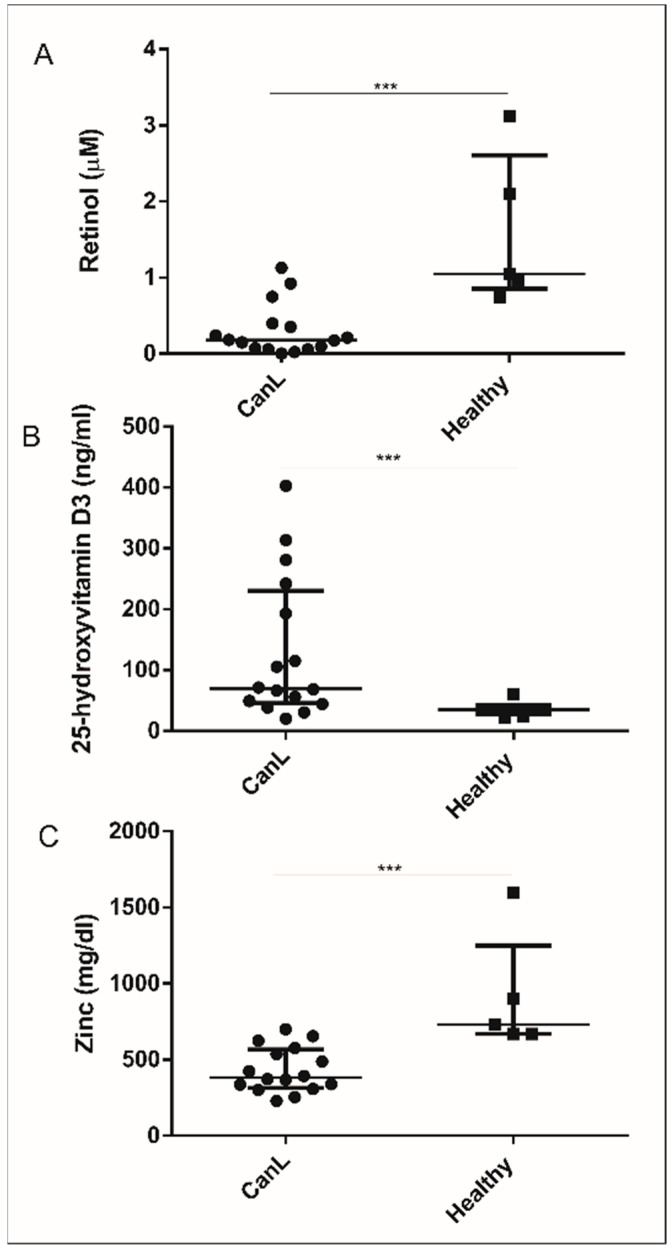
Serum levels of nutrients in the CanL group and in healthy dogs. Low levels of retinol (**A**) and Zn (**C**) and higher levels of 25(OH)VD_3_ (**B**) were observed in the CanL group (*n* = 15) compared to healthy dogs (*n* = 5). Data represent the median and interquartile range (IQR). Asterisks indicate a significant difference between the CanL group and healthy dogs (Mann–Whitney test, *** *p* < 0.001).

Vitamin D deficiency has been associated with the progression of CanL [19], and low serum levels of vitamin A [20] and Zn [27] have been detected in patients with leishmaniasis. On this basis, we quantitated the serum levels of these nutrients in the study dogs and observed low levels of retinol (Figure 1A) and Zn (Figure 1C) in the CanL group compared to healthy dogs, while there were higher levels of 25(OH)VD_3_ in the CanL group (Figure 1B). 

### 3.2. In Vitro Supplementation of Spleen Leukocytes with ATRA, 1,25(OH)_2_VD_3__,_ and SZn Increased Production of NO and ROS in the CanL Group Stimulated with SLA

**Figure 2:**
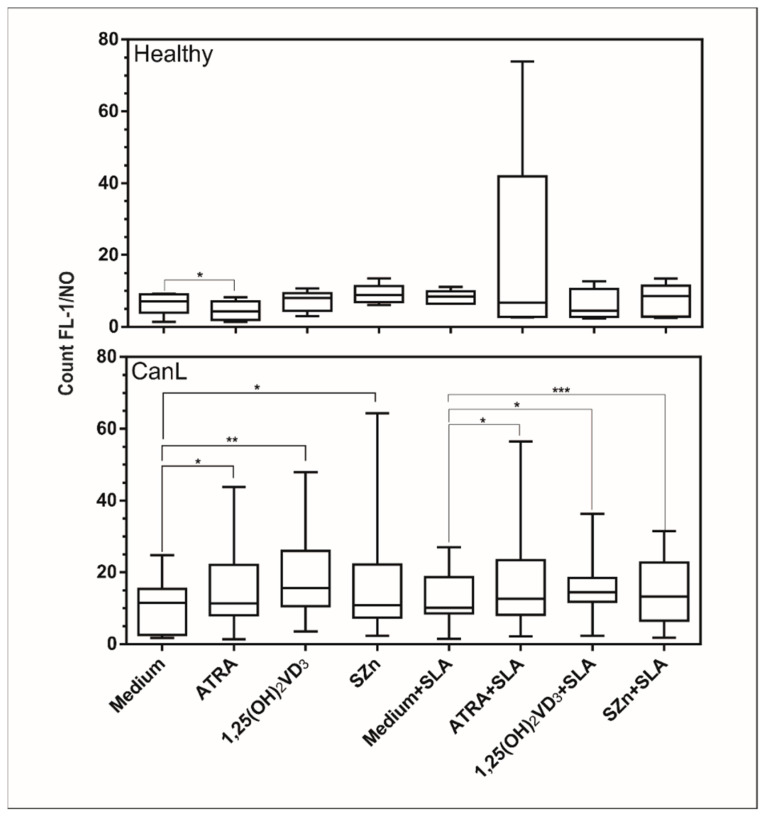
NO production by spleen leukocytes of dogs with CanL and healthy dogs supplemented with nutrients. Spleen leukocytes from CanL (*n* = 15) and healthy dogs (*n* = 5) were cultured in the medium alone or with the addition of ATRA, 1,25(OH)_2_VD_3_ and SZn, with and without SLA. NO production in the cell culture was determined by flow cytometry after 20 h. Bars represent the median and IQR with minimum and maximum error bars. Asterisks indicate a significant difference for the comparisons within the treatment (Wilcoxon test, * *p* < 0.05, ** *p* < 0.01, and *** *p* < 0.001).

**Figure 3:**
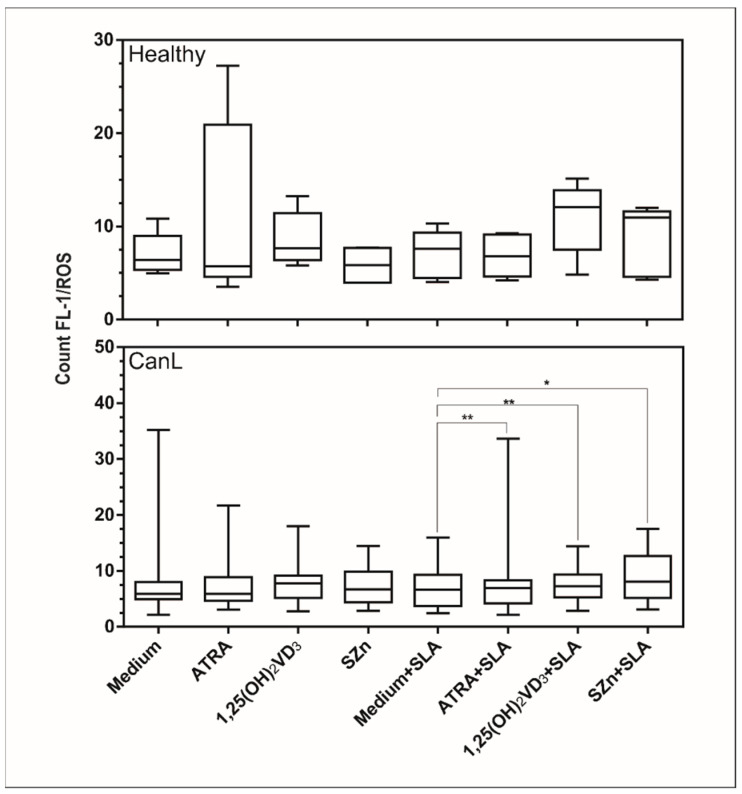
ROS production by spleen leukocyte cultures of the dogs with CanL and healthy dogs supplemented with nutrients. Spleen leukocytes from CanL (*n* = 15) and healthy dogs (*n* = 5) were cultured in the medium alone or with the addition of with ATRA, 1,25(OH)_2_VD_3_ and SZn, both with and without SLA. ROS production in the cell cultures was determined by flow cytometry after 20 h. Bars represent the median and IQR with minimum and maximum error bars. Asterisks indicate a significant difference for the comparisons within the treatment (Wilcoxon test, * *p* < 0.05 and ** *p* < 0.01).

The reactive species NO and ROS are important for leishmanicidal activity [28], NO having been previously demonstrated in CanL [29]. On this basis, we determined the production of NO and ROS in spleen leukocytes of the CanL group after in vitro supplementation with the nutrients and observed an increased production of NO (Figure 2) in the spleen leukocytes of the CanL group with ATRA, 1,25(OH)_2_VD_3_, and SZn but no change in the ROS production (Figure 3). The addition of SLA with ATRA, 1,25(OH)_2_VD_3_, and SZn increased both the NO (Figure 2) and ROS (Figure 3) production in cells. In the control group, the NO production was increased in splenic leukocytes only after the addition of ATRA (Figure 2), whereas no effect was observed for the remaining treatment conditions or for the ROS production (Figure 3). 

### 3.3. In Vitro Supplementation of Spleen Leukocytes with 1,25(OH)_2_VD_3_ and SZn Reduced IFN-γ; ATRA and SZn Increased TNF-α; and ATRA, 1,25(OH)_2_VD_3_, and SZn Reduced IL-10 in the CanL Group Stimulated with SLA

**Figure 4:**
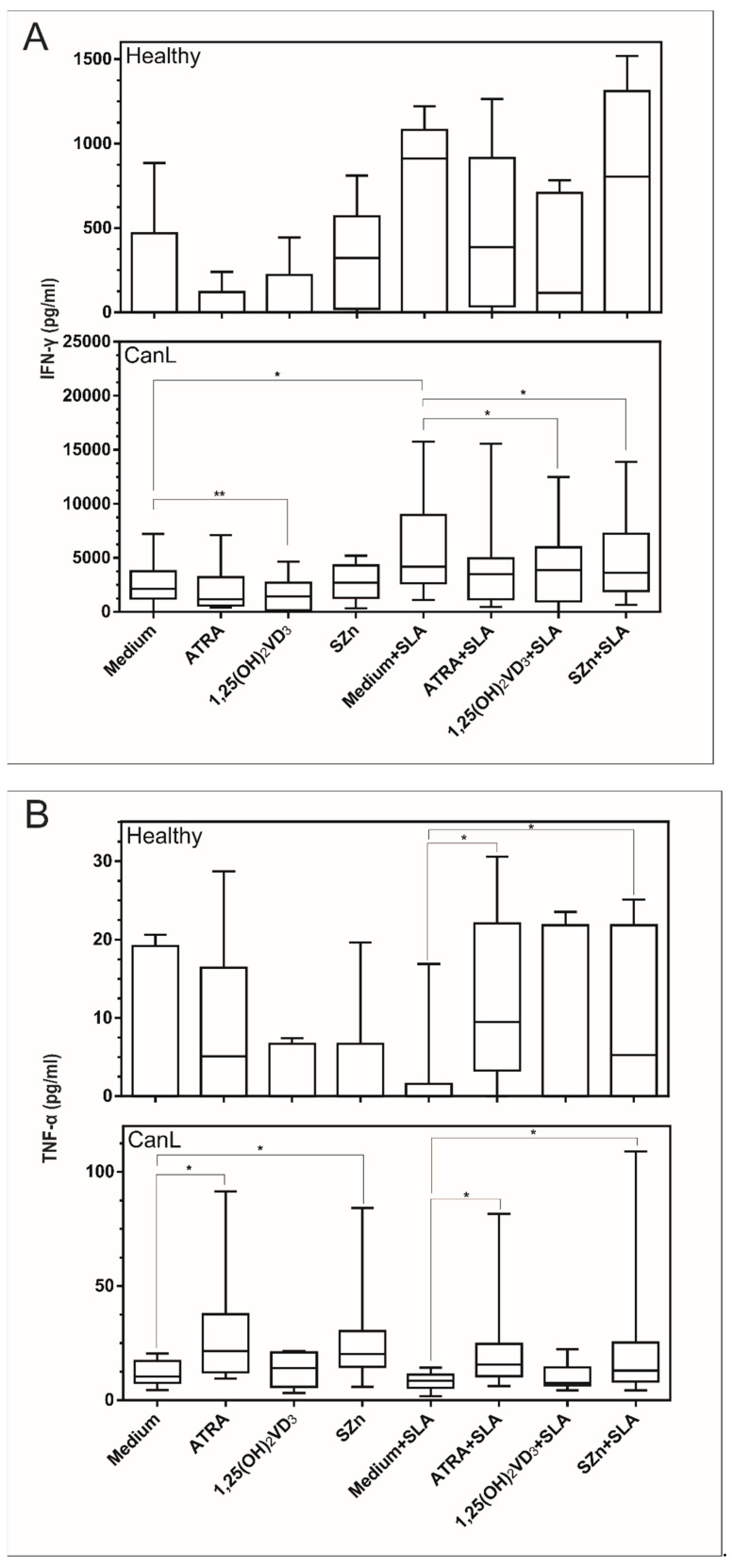

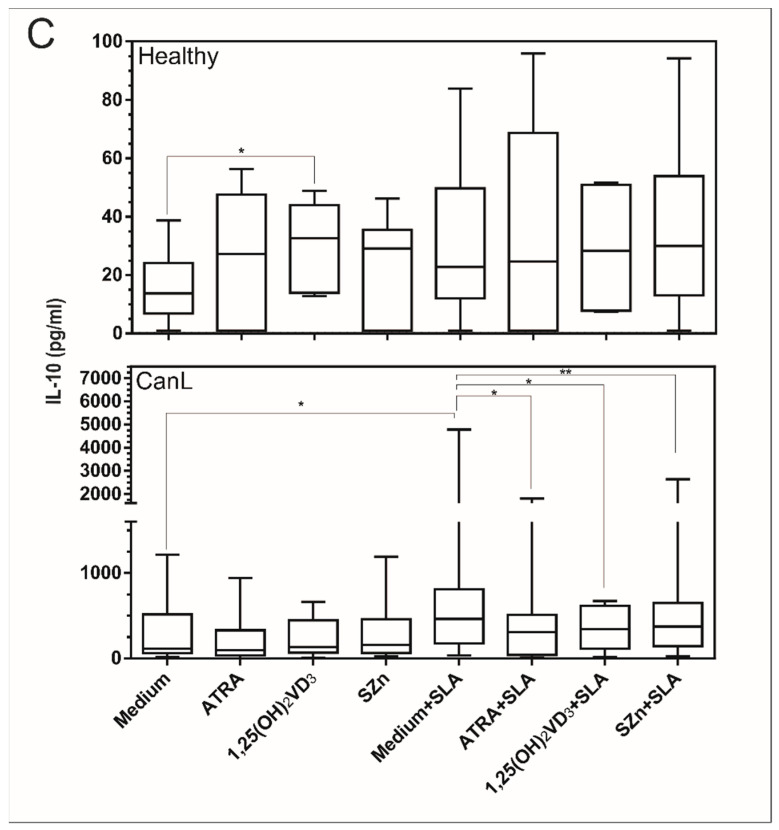
Cytokine production in the supernatant of spleen leukocyte cultures of the CanL group and the control group of healthy dogs supplemented with nutrients. Spleen leukocytes from the CanL (*n* = 10, 7 and 15 for INF-γ, TNF-α and IL-10, respectively) and healthy dogs (*n* = 5) were cultured in medium alone or with the addition of ATRA, 1,25(OH)_2_VD_3_ and SZn, with and without SLA. After 72 h, IFN-γ (**A**), TNF-α (**B**) and IL-10 (**C**) production in the culture supernatant was determined by ELISA. Bars represent the median and IQR with minimum and maximum error bars. Asterisks indicate a significant difference for the comparisons within the treatment (Wilcoxon test, * *p* < 0.05 and ** *p* < 0.01).

We also determined whether in vitro supplementation with the above nutrients would interfere with the production of immunity regulating cytokines in CanL. The supplementation of spleen leukocytes with PHA led to high IFN-γ, TNF-α, and IL-10 production in the culture supernatant for all groups and treatments, confirming that the assay worked (data not shown). We observed that IFN-γ (Figure 4A) was increased in comparison to the medium alone and the medium with SLA and was reduced in the culture supernatant of the CanL group following the treatment with 1,25(OH)_2_VD_3_ but not after the treatment with ATRA or SZn. IFN-γ (Figure 4A) was also significantly reduced in the culture supernatant of the CanL group after the spleen leukocyte stimulations with SLA, 1,25(OH)_2_VD_3_, and SZn. No effect was observed for ATRA in terms of IFN-γ production (Figure 4A). In the control group, the supplementation of spleen leukocytes with ATRA, 1,25(OH)_2_VD_3_, and SZn did not interfere with the production of IFN-γ under any of the treatment conditions studied (Figure 4A). 

TNF-α was significantly increased in the culture supernatant of the spleen leukocytes stimulated with ATRA and SZn in the CanL and control groups in the presence of SLA (Figure 4B), but in the absence of SLA, only the CanL group exhibited this effect. No effect was observed with the remaining treatment conditions or for the stimulation with 1,25(OH)_2_VD_3_ (Figure 4B).

IL-10 was significantly increased in the medium alone when compared to the medium and SLA (Figure 4B), and it was reduced in the culture supernatant of the spleen leukocytes stimulated with ATRA, 1,25(OH)_2_VD_3_, and SZn in the CanL group in the presence of SLA, while no effect was observed in the absence of SLA. In the control group, IL-10 production was increased in the spleen leukocytes only after the addition of 1,25(OH)_2_VD_3_. No effect was observed for the remaining treatment conditions (Figure 4C). 

### 3.4. In Vitro Supplementation of Spleen Leukocytes in the CanL Group Stimulated with 1,25(OH)_2_VD_3_ and SZn Reduced the Parasite Load, but after the Stimulation with SLA, No Effect Was Observed

**Figure 5:**
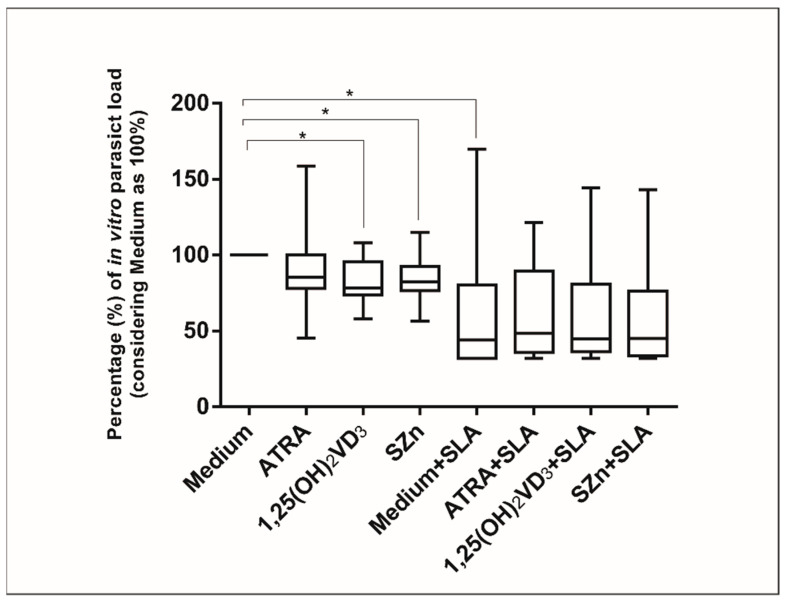
Parasite load in the spleen leukocytes of the CanL group supplemented with nutrients. Spleen leukocytes from dogs with CanL (*n* = 15) were cultured in the medium alone or in medium supplemented with ATRA, 1,25(OH)_2_VD_3_ and SZn, either with or without SLA. After 72 h, the parasite load was quantitated by counting the amastigotes present inside the infected macrophages on slides obtained by cytocentrifugation and was determined by dividing the number of amastigotes by the number of infected macrophages counted. The results are expressed as a percentage (%), considering the medium as 100% in relation to the other treatments. Bars represent the median and IQR with minimum and maximum error bars. Asterisks indicate a significant difference for the comparisons within the treatment (Wilcoxon test, * *p* < 0.05).

Intracellular Zn can influence the phagocytosis and microbicidal activity [24]. On this basis, we determined whether the supplementation of a spleen leukocyte culture of the CanL group with ATRA, 1,25(OH)_2_VD_3_, and SZn would affect the parasite load. The parasite load was reduced by the nutrient supplementation in comparison with the medium alone, the medium with SLA, and the medium supplemented with 1,25(OH)_2_VD_3_ and SZn in the absence of SLA. However, in the presence of a stimulation with SLA, no effect of these nutrients was observed (Figure 5). 

## 4. Discussion

In this study, we observed low retinol and Zn levels and increased 25(OH)VD_3_ levels in the serum of dogs with leishmaniasis compared to healthy dogs. The in vitro supplementation of CanL spleen leukocytes with ATRA, 1,25(OH)_2_VD_3_, and SZn, in addition to the treatment with SLA, caused an increase in NO and ROS production, while ATRA and SZn increased the TNF-α levels. Increased IL-10 and IFN-γ levels were observed with the addition of SLA to the medium. However, when the three nutrients were added, there was a significant reduction of the IL-10 levels, and with the addition of 1,25(OH)_2_VD_3_ and SZn, there was a significant reduction of IFN-γ. The supplementation with 1,25(OH)_2_VD_3_ and SZn reduced the parasite load, whereas, in the presence of the stimulation with SLA, no effect was observed for these nutrients or for ATRA. These findings suggest an important role of these immunomodulatory nutrients in CanL, with positive effects that favor an effector Th1 response.

The low serum level of retinol observed in dogs with leishmaniasis confirmed the previous results regarding this nutrient in the disease, such as a low serum vitamin A level in children with visceral leishmaniasis in an endemic area of Brazil [20]. A vitamin A deficiency affects the immunity of mice, reduces the number of Th1 memory cells [30], and provokes an abnormal expansion of myeloid cells [31]. In symptomatic dogs, the histopathological changes associated with the increased myeloid cell levels in the spleen [32] may be related to the low serum level of retinol observed here in the dogs with CanL.

The low serum Zn levels we observed in dogs with leishmaniasis confirmed the previous findings reported for dogs [33,34,35] and for humans with chronic visceral leishmaniasis living in endemic areas of India [36] and Bangladesh [37]. Dogs with leishmaniasis from an endemic area of Western Turkey had reduced Zn levels in their serum and hair compared to a control group, although this difference was statistically significant only for the serum levels [35]. A Zn deficiency has effects on immunity, such as the reduction of peritoneal macrophages in mice and a lower microbicidal activity of these cells, while a Zn addition restores the ability of macrophages to capture and kill the parasite *Trypanosoma cruzi* [38]. In addition, Zn is essential for thymus function, with atrophy of the organ occurring under Zn deficiency, as well as lymphopenia and depression of both the immune and adaptive response [18,39]. The adaptive response is suppressed in dogs with leishmaniasis [2], and part of this suppression may be related to a Zn deficiency. Furthermore, the Zn status influences the vitamin A status, because it is involved in several processes, such as vitamin A absorption, transport, and utilization [40]. Furthermore, vitamin A [41] and Zn [18] are antioxidants, and their use in the fight against the oxidative stress caused by leishmaniasis [42] may be at the root of the observed deficiency in this disease. Since vitamin A and Zn deficiencies have been extensively reported in leishmaniasis, we emphasized the need for supplementation with these nutrients as a preventive strategy or as a coadjutant treatment in view of their importance to the immune system.

We observed high serum vitamin D levels in dogs with leishmaniasis. This contrasts with the results of a cohort of 68 dogs from an endemic area in Spain, whose serum vitamin D levels were lower in animals with leishmaniasis compared to uninfected and asymptomatic animals, and this vitamin D deficiency was thought to be associated with disease progression [19]. This difference may be explained by the high incidence of solar radiation in Brazil compared to Spain, since most vitamin D is produced on the skin by exposure to UVB radiation [43]. In addition, our control group consisted of dogs belonging to private owners who usually reside inside their homes protected from the sun, possibly explaining the low serum 25(OH)VD_3_ levels of this group. In addition, Zn deficiency, by changing the activity of VDR, modulates the protein expression of VDR [44,45] and may have caused the high vitamin D status in the serum that we observed. 

The supplementations with all three nutrients tested in the present study, in the presence or absence of SLA, increased the NO levels in the spleen leukocytes of the CanL-affected dogs, although these nutrients only induced an increase in ROS production when the cells were stimulated with SLA. NO and ROS exhibit microbicidal activity in canine leishmaniasis [9,28,46], suggesting that these micronutrients may potentiate the leishmanicidal effect of the Th1 response. It has been demonstrated that the inhibition of ROS production in human monocytes from patients with cutaneous leishmaniasis caused by *L. braziliensis* permitted the growth of viable promastigotes in a culture supernatant, whereas NO was associated with the lesion size [9]. The authors confirmed the leishmanicidal activity of ROS and observed that NO alone does not control the infection and seems to contribute to tissue damage [9].

The in vitro increase in NO in the spleen leukocytes of CanL-affected animals supplemented with ATRA may have been due to activation of the promoter region of the induced nitric oxide synthase (iNOS) gene, as previously demonstrated in vitro in humans [47] and in vivo in mice [48]. In fact, we found in this study a putative response element (RE) for RAR/RXR in the proximal promoter region of the canine iNOS by performing an in silico promoter analysis using the Nsite online tool [49] with the RegsiteAN database [50]. The predicted RE is located within 100 bps of the predicted transcriptional start site. In agreement with our results, the addition of 1,25(OH)_2_VD_3_ also increased the NO production in murine macrophages activated by IFN-γ and infected with *L. major* [26]. Future studies are needed to elucidate the mechanism by which ATRA, 1,25(OH)_2_VD_3_, and Zn increase the NO levels in dogs with leishmaniasis.

We also investigated the cytokine levels of the spleen leukocytes from CanL animals after supplementation with ATRA, 1,25(OH)_2_VD_3_, and SZn and the presence and absence of SLA. In vitro supplementation with 1,25(OH)_2_VD_3_ reduced the IFN-γ levels in the cell culture supernatant, an effect that was not observed with ATRA or SZn. In the presence of SLA, 1,25(OH)_2_VD_3_ and SZn also reduced the IFN-γ levels in the supernatant of the leukocyte culture, whereas ATRA had no effect. Despite being a Th1 response cytokine, high levels of IFN-γ have been reported in symptomatic dogs with CanL, as well as even higher levels of IL-10, and are associated with a high spleen parasite load and worsening of the disease [51,52,53], and the reduction of IFN-γ observed in the present study may have been favorable to the regulation of effector immunity, since we also detected a low parasite load in the presence of 1,25(OH)_2_VD_3_ and SZn. 

Increased IFN-γ in the supernatant of spleen leukocytes was observed in dogs with CanL when comparing the medium alone with the medium in the presence of SLA. This may be associated with activation of the Th1response. The reduction of IFN-γ in the supernatant of spleen leukocytes observed in the CanL group after supplementation with 1,25(OH)_2_VD_3_ confirmed the anti-inflammatory role of vitamin D, which was also observed in experimental leishmaniasis [54,55] and in other infectious parasitic [56,57] and autoimmune diseases [58].

ATRA and SZn increased the TNF-α levels in the cell culture supernatant with or without the use of SLA, while 1,25(OH)_2_VD_3_ had no effect. TNF-α is associated with the effector Th1 response in the fight against disease [59], and its increased levels after supplementation with SZn agree with the results of an in vitro study using peripheral blood mononuclear cells (PBMC) from healthy humans [60]. Regarding the effect of ATRA on the increased levels of TNF-α, we suggest a positive effect on the Th1 response of dogs with leishmaniasis, in contrast to the results obtained in vitro in mouse PBMC, in which the supplementation with ATRA reduced the TNF-α mRNA, a difference that may be related to the different host and experimental designs used in the studies. The modulation of TNF-α production by ATRA and SZn suggests that these nutrients may regulate the adaptive response of the Th1 cell profile.

In this study, the production of IL-10 was increased in the supernatant of spleen leukocytes of the CanL-affected dogs when SLA was supplemented compared to the medium alone, but this effect was reduced when the SLA supplementation was combined with all three nutrients. In agreement with our results, the in vitro supplementation with ATRA of PMBC (Treg and monocytes) from humans infected with *L. infantum* reduced the expression of IL-10 [20], indicating a possible regulatory role of vitamin A (down modulate) in the production of IL-10 in visceral leishmaniasis. The IL-10 cytokine in the spleen seems to be an indicator of leishmaniasis susceptibility [61], and its reduction observed in the CanL group after supplementation with ATRA, 1,25(OH)_2_VD_3_, and SZn may favor the regulation of immunity. 

Monocytes or macrophages are not just the primary host cells for leishmania but also the main cells with the ability to eradicate parasites [9]. Thus, the parasite load was reduced with SLA supplementation, as compared to the medium alone, possibly due to the activation of the Th1 response by the addition of antigens. In addition, the supplementation with 1,25(OH)_2_VD_3_ and SZn reduced the parasite load, as shown by the count of infected macrophages, although no effect was observed when the same preparation was combined with the SLA stimulation. In agreement with these results, mice infected with *L. donovani* and receiving a diet enriched with ATRA and 1,25(OH)_2_VD_3_ also showed a reduced spleen parasite load [21]. Although the difference was not statistically significant, there was a numerical tendency to a reduction in the parasite load with the addition of SLA and the three nutrients studied here when compared with the medium alone. The limiting factors, such as the sample size and high data variability, which are characteristic of a canine population, may explain the lack of statistical significance. Two other important limitations of this study should be highlighted: There was a lack of vehicle control groups used in the preparation of the nutrients, and we lacked data regarding the diet of the dogs included in the study, as we know that diet can influence the in vitro results and the serum levels of nutrients. In addition, Zn supplementation has proven to be beneficial in various infectious diseases, including leprosy and tuberculosis, among others [62]. The therapeutic response of dogs with leishmaniasis treated with standard therapy in combination with Zn was more rapid, with a longer interval before relapse [62], supporting the important role of this mineral in the disease. These findings suggest that the ATRA, 1,25(OH)_2_VD_3_, and SZn nutrients may be linked to the leishmanicidal activity of macrophages, although further studies are needed to elucidate the mechanisms involved.

## 5. Conclusions

In summary, we showed that canine leishmaniasis is related to vitamin A and Zn deficiency, and we suggest that ATRA, 1,25(OH)_2_VD_3_, and SZn are involved in the immunological reaction associated with the leishmanicidal effector response, with great potential for further investigations using an in vivo model.

## Data Availability

The data presented in this study are available on request from the corresponding author.
